# Lignans from *Sambucus williamsii* Protect Bone Via Microbiome

**DOI:** 10.1007/s11914-024-00883-1

**Published:** 2024-09-05

**Authors:** Hui-Hui Xiao, Daniel Kam-Wah Mok, Xin-Sheng Yao, Man-Sau Wong

**Affiliations:** 1 Department of Food Science and Nutrients, The Hong Kong Polytechnic University, Hong Kong, China; 2 Research Centre for Chinese Medicine Innovation, The Hong Kong Polytechnic University, Hong Kong, China; 3 State Key Laboratory of Chinese Medicine and Molecular Pharmacology (Incubation), The Hong Kong Polytechnic University Shenzhen Research Institute, Shenzhen, 518057 China; 4 Institute of Traditional Chinese Medicine & Natural Products, Jinan University, Guangzhou, China

**Keywords:** Osteoporosis, lignans, gut microbiota, serotonin synthesis

## Abstract

**Purpose of Review:**

Traditional Chinese Medicine has a unique system to diagnose and treat bone diseases with symptoms similar to those of osteoporosis. *Sambucus williamsii* Hance (SWH), a folk medicine in northern part of China for fractures healing and pain alleviation, has been demonstrated to exert bone anabolic effects in ovariectomized (OVX) rat and mice models in our previous studies. Lignans were identified to be the main bioactive fractions of SWH. However, pharmacokinetics study showed that the levels of lignan were too low to be detected in rat serum even upon taking 15 times of the effective dose of lignan-rich fraction from SWH. We hypothesize that lignans from SWH might exert its bone protective effect via the gut microbiome.

**Recent Findings:**

Our study revealed that the lignan-rich fraction of SWH did not influence the diversity of gut microbiota in OVX rats, but significantly increased the abundance of a few phyla, in particular, the restoration of the abundance of several genera that was directly correlated with bone mineral density (BMD). In addition, a subsequent metabolomic study indicated that serotonin, a neurotransmitter synthesized in the intestine and influenced by gut microbiota, may be involved in mediating the bone protective action of the lignans. Gut-derived serotonin is thought to inhibit bone growth. Based on this finding, several inhibitors that suppressed the synthesis of serotonin were identified from the lignans of SWH.

**Summary:**

Our studies suggested that microbiome is an indispensable factor for lignans derived from *S. willimasii* to exert bone beneficial effects.

## Lignans and its Role on Bone Health

The proportion of the world's population over 60 years will be nearly double from 12 to 22% between 2015 to 2050 [1]. It is anticipated that 21.2% of women and 6.3% of men globally will suffer from osteoporosis and might lead to almost 70 fragility fracture per minute [2]. Hormonal changes, especially estrogen deficiency associated with menopause, and aging are the major contributing factors to the pathogenesis of osteoporosis.

Phytoestrogens, which are classically defined as phytochemicals that possess similar chemical structures to endogenous estrogen, are considered to be potential alternatives to hormone replacement therapy for management of estrogen deficiency-induced symptoms in menopausal women [3]. Lignans, one of the three major classes of phytoestrogens, are widely present in a variety of plant foods, including seeds, beans, whole grains, bran, fruits, vegetables and beverages such as tea and coffee [4]. Although the epidemiological studies are inconclusive, numerous health benefits have been attributed to the intake of naturally lignan-rich foods, including the reduction of risk of heart disease, menopausal symptoms, breast cancer and osteoporosis [3, 5].

Flaxseed and sesame seeds, derived from *Linum usitatissimum L.* and *Sesamum indicium*, respectively, are the two richest known sources of lignans. The consumption of flaxseed in various forms including total extract, power, flour, and oil, exhibited positive effects on bone mineral density (BMD) and bone metabolism in different animal models and in several clinical studies [6]. From 2013 to 2022, six studies reported that sesame seeds intake only, or in combination with peanut seeds or black seeds, were beneficial for improving BMD and serum bone biochemical markers in postmenopausal animal models [7]. In contrast, a randomized, double-blind clinical trial including 199 healthy menopausal women consuming 40 flaxseed/d (n = 101) or wheat germ placebo (n = 98) for 12 months, revealed that flaxseed incorporation into the diet of healthy menopausal women did not alter the BMD in lumbar or femoral neck as compared with wheat germ placebo [8]. Due to the limited available clinical studies, the effects of lignans on bone health are inconclusive.

## Lignan Bioavailability and Correlation with Gut Microbiota

Several early studies revealed that ingested lignans were deglycosylated and converted to mammalian lignans enterolactone and enterodiol by gut microbiota, absorbed into blood, finally excreted in urine, bile and feces [9]. Lignans such as pinoresinol, lariciresinol, secoisolariciresinol diglucoside (SDG) and matairesinol which represented four different classes of lignans were converted to enterolignans through reduction, O-deglycosylation, O-demethylation, dehydrogenation and dihydroxylation by intestine bacteria [10]. However, only 55%, 62% and 72% of pinoresinol diglucoside, matairesinol and SDG were converted to enterolignans, respectively(11). A study showed that one hour after ingestion of sesame seeds, some lignans are quickly detected in the systemic circulation, such as pinoresinol, matairesinol, lariciresinol, secoisolariciresinol and sesamin, while other studies indicated that the enterolignans generally appeared in circulation 8–10 h after intake of dietary lignans [9]. Sacco et al. reported that after orally administering rats with trimer-labeled SDG for 7 days, 59.8% of the radioactivity, including the prototype and metabolites, was detected in the colon and cecum, while only 0.1% was detected in the entire skeletal tissue [12]. In addition, the fate of pure compounds does not invariably mirror the bioavailability of lignan within complex food or herbal medicines matrices. Therefore, the above publications suggest that the enterolignans or metabolites of lignans in the circulation might not be able to totally account for the beneficial actions of lignans on bone.

## A Lignan-Rich Herb *Sambucus willamsii* Hance and its Effects on Bone

*Sambucus williamsii* Hance (SWH) belongs to *Caprifoliaceae* family and is widely distributed in China, Korea and Japan [13]. It is in the same genus as elderberry (also known as *Sambucus* nigra) which is popular in Europe to be used in products of syrup, gummies, soft drink, liqueur and eye gel. *S. williamsii* was first recorded in *Tang Materia Medica* (A.D. 659) for healing fractures and alleviating pain. Our previous studies have demonstrated that the SWH extract and its lignan-rich fraction increased BMD, improved trabecular bone micro-architecture, and promoted cortical bone strength in ovariectomized mouse and rat models [14–17]. Fifty-six lignans were isolated and purified in the extract of SWH and were identified to be the bioactive components in SWH through the determination of the osteoblastic proliferation in vitro [18, 19].

### A Question Arises from Pharmacokinetics Study

In order to identify the major bioavailable lignans that are associated with the bone protective effects of SWH, a pharmacokinetic study using rat model was carried out. The rats were administrated with an effective dose or a 15-times effective dose of the SWH fraction, followed by collection of the blood samples at 0, 5, 15, 30, 60, 120, 180, 240, 360, and 480 min. However, neither the prototype nor metabolites were detected by highly sensitive method using an ultra-high performance liquid chromatography with quadrupole time-of-flight mass spectrometry (UPLC-QTOF/MS). To accumulate sufficient exogenous components in blood, samples were again collected and analyzed from rats gavaged daily with effective dose or 15-times effective dose of SWH fraction for 3 days. However, no detectable prototype nor metabolites of lignans could be detected in serum collected from these rats treated with SWH fraction for 3 days. The in-house results indicated that lignan-rich fraction may be composed of too many lignans with too low concentration of each type of lignan to be detected in blood. However, the lack of detectable lignans in the circulation might also suggest that mechanisms that do not require gastrointestinal uptake of lignans might be involved. This unsuccessful pharmacokinetic study prompted us to explore other mechanisms that might account for the bone protective effects of lignans.

### Gut Microbiome Interaction with Lignan

Based on the fact that the bone protective effects of SWH was demonstrated in response to its oral administration, we hypothesized that lignans from SWH might exert its bone protective effect via the gut microbiome, including gut microbiota, their metabolites and their living environment [20]. An animal study using the lignan-rich fraction (CA) from SWH was performed to test the hypothesis [21]. Female SD rats (4-month old) were sham operated or ovariectomized, and treated with vehicle, PTH (1.8 µg/kg, n = 8, intramuscularly injected), CA (140 mg/kg, n = 10) for 10 weeks. The results exhibited that the lignan-rich fraction from SWH at 140 mg/kg prevented the reduction in BMD at distal femur and proximal tibia and improved bone microarchitecture in OVX rats, and these effects were similar to those of PTH on the trabecular bone.

The 16S rRNA sequencing of fecal samples showed that the microbial diversities of all groups increased significantly upon 10 weeks of treatment when compared to OVX group at 0 week, while no significant differences were found among the Sham, OVX and CA groups at week 10. The Principal Coordinate analysis (PCoA) and Partial least squares discriminant analysis (PLS-DA) analysis showed that the operational taxonomic units (OTUs) at week 10 were well separated from that at week 0, suggesting the microbial composition had altered over time. At week 10, the bacteria in the CA treatment group induced a significant separation from that of the Sham and OVX groups, indicating that CA treatment had significant influence on intestinal bacterial composition.

The difference analysis between groups and the LEfSe (linear discriminant analysis Effect Size) analysis showed that the abundance of *Antinobacteria* phylum extremely enriched in OVX rat fecal samples in response to CA treatment, and the taxa contributed to the abundance included: class, *Antinobacteria*; order, *Coriobacteriales*; family, *Coriobacteriacene*; genus, *Adlercreutzia*, norank_f_*Coriobacteriaceae*, *Parvibacter*, *Enterorhabdus*, *Collinsella*. The five genera belonged to *Antinobacteria* phylum were significantly reversed in the OVX rats treated with CA when compared with the OVX groups. These altered genera are related to the production of short chain fatty acid, modification of host bile acids and cholesterol levels, low dietary fiber intake and circulating insulin, cholesterol absorption, synthesis, and excretion [21]. Further spearman’s correlation analysis revealed that BMD was directly correlated with several genera, such as *Lachnospiraceae_NK4A136_group* and *[Eubacterium] coprostanoligenes group,* which are related to tryptophan metabolism. The results indicated that CA treatment might indirectly affect bone metabolism via modifying the metabolism of tryptophan in the gut.

### Serotonin Involved in the Actions of Lignans on Bone

A metabolomic study was performed to identify if tryptophan metabolism was involved in the actions of lignans on bone. The resulted showed that tryptophan level in OVX rats was significantly restored upon treatment with the lignan-rich fraction of SWH for 12 weeks [17]. Tryptophan has two major catabolic pathways, the kynurenine (Kyn) pathway and serotonin (5-HT) pathway. The serum kynurenine level and serotonin level upon treatment with CA were quantified using an ultra-performance liquid chromatography tandem triple quadrupole mass spectrometry (UPLC-TQD-MS) method. The results showed that CA treatment significantly reduced serum 5-HT level, but not Kyn level, in OVX rats. The linear regression analysis showed that BMD was negatively correlated to serum 5-HT in rats [21].

Serotonin is best known as a neurotransmitter, and more than 90% of the body’s serotonin is synthesized by enterochromaffin cells in intestine, while the others is synthesized by clusters of neurons in brain. As serotonin cannot pass through the blood–brain barrier, all serotonin in the circulatory system comes from intestine. Tryptophan hydroxylase (TPH) is the rate-limiting enzyme of 5-HT biosynthesis, and only the subtype of TPH-1 is located in the intestine, while the subtypes of TPH-1 and TPH-2 are distributed in the brain [22]. Our studies showed that CA significantly suppressed the protein expression of TPH-1 in the colon, while did not alter the protein expressions of TPH-1 and TPH-2 in cerebral cortex in OVX rats.

For the serotonin signaling pathway in osteoblast, the gut-derived 5-HT is delivered to osteoblasts by platelets, and its binding to the membrane 5HT1b receptor will then trigger the FOXO1/CRBE and FOXO1/ATF4 signaling cascade in osteoblastic cells. When high level of 5-HT acts on osteoblasts, the balance between FOXO1/CRBE and FOXO1/ATF4 will be broken and lead to the decrease in bone formation [23]. In our study, the mRNA and protein expressions of CREB in femur were not altered by OVX nor CA treatment. In contrast mRNA and protein expressions of ATF4 and FOXO1 in femur were significantly upregulated and downregulated in OVX rats, respectively; and these changes were significantly reversed in OVX rats in response to treatment with CA [21].

Taken together, our study clearly demonstrated that the lignan-rich fraction derived from *Sambucus williamsii* Hance exerted bone protective effects via altering circulating serotonin and gut microbiota.

### Exploring a Novel Class of TPH-1 Inhibitor

Based on our discovery of the involvement of TPH-1 and serotonin in mediating the actions of lignan-rich fraction of SWH, subsequent study to identify TPH-1 inhibitors from SWH was performed [24]. The binding affinities of lignans isolated and purified from SWH were determined by molecular docking and surface plasmon resonance (SPR), the protein activity and expression of TPH-1 was tested in vitro, and serum serotonin level and BMD in mice upon treatment with purified lignan compound were measured. Our results showed that lignans derived from SWH exhibited high binding affinities to the TPH-1 protein, inhibited the activity and expression of TPH-1 in RBL2H3 cells, the *Tph-1* gene high-expressing cells, and suppressed serum serotonin levels as well as significantly improved BMD in OVX mice. A series of novel class TPH-1 inhibitors, the lignans derived from SWH, were explored. The results provided the direct evidence that lignans could act on TPH-1 and influence serotonin synthesis and its circulating levels, thereby indirectly modulate bone metabolism in OVX animal model.

## Conclusion

**Fig. 1:**
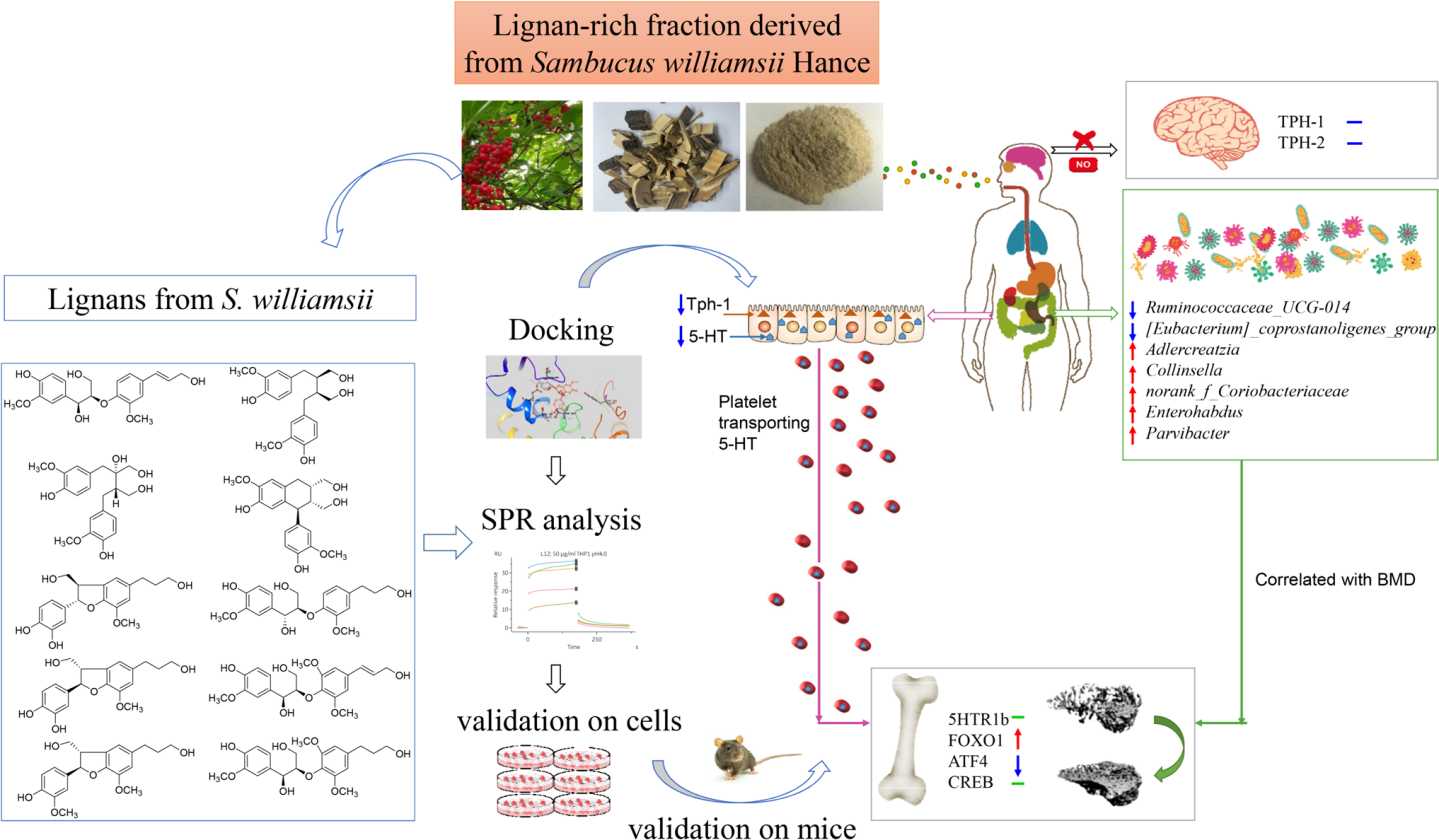
The action scheme of lignan-rich fraction derived from *Sambucus williamsii* Hance one bone

The mechanism of actions of lignan derived from SWH were summarized in Fig. 1. In conclusion, lignans appear to exert protective effects on bone by modulating the composition of microbiota and suppressing the synthesis of gut-derived serotonin, without the need to be absorbed into the circulation. Microbiome, including microbiota, their metabolites and their living environment, is an indispensable factor for lignans to exert bone beneficial effects.

## Key References

- International Osteoporosis Foundation. Epidemiology of osteoporosis and fragility fractures. https://www.osteoporosis.foundation/facts-statistics/epidemiology-of-osteoporosis-and-fragility-fractures: International Osteoporosis Foundation. 2024.
  - Overview the latest epidemiology of osteoporosis and fragility fractures worldwide, along with the burden and impact of hip fractures and vertebral fractures, and outlined the rate of fractures caused by falls and the risk of secondary fractures.
- Batool I, Altemimi AB, Munir S, Imran S, Khalid N, Khan MA, et al. Exploring flaxseed's potential in enhancing bone health: Unveiling osteo-protective properties. Journal of agriculture and food research 2024; 15: 101,018.
  - Comprehensive overview of the bone protective efficacy of flaxseed in different forms like oil, flour, extract and powder in preclinical experiments and clinical trials, and revealed the mechanism of promoting bone health and density by unsaturated fatty acid, lignans and fiber in flaxseeds.
- Arooj A, Rabail R, Naeem M, Goksen G, Xu B, Aadil RM. A comprehensive review of the bioactive components of sesame seeds and their impact on bone health issues in postmenopausal women. Food Funct 2023;14(11):4966–498.
  - Good summary of the positive role of sesame seeds on bone health in postmenopausal women and animal models, and the nutritional composition and bioactive ingredients of sesame seeds. Supported the use of sesame seeds as supplements in regular diet.

## Data Availability

No datasets were generated or analysed during the current study.
